# Preparation of therapy-grade extracellular vesicles from adipose tissue to promote diabetic wound healing

**DOI:** 10.3389/fbioe.2023.1129187

**Published:** 2023-03-23

**Authors:** Chuqiao Pan, Peng Xu, Yi Zheng, Yikai Wang, Chuhsin Chen, Shibo Fu, Zibo Liu, Yahong Chen, Ke Xue, Qimin Zhou, Kai Liu

**Affiliations:** Department of Plastic and Reconstructive Surgery, Shanghai Key Laboratory of Tissue Engineering, Shanghai Ninth People’s Hospital, Shanghai Jiao Tong University School of Medicine, Shanghai, China

**Keywords:** adipose tissue, extracellular vesicles, wound healing, diabetes, therapy-grade

## Abstract

**Background:** Treatment of diabetic wounds is a major challenge in clinical practice. Extracellular vesicles (EVs) from adipose-derived stem cells have shown effectiveness in diabetic wound models. However, obtaining ADSC-EVs requires culturing vast numbers of cells, which is hampered by the need for expensive equipment and reagents, extended time cost, and complicated procedures before commercialization. Therefore, methods to extract EVs from discarded tissue need to be developed, for immediate application during surgery. For this reason, mechanical, collagenase-digestive, and constant *in-vitro*-collective methods were designed and compared for preparing therapy-grade EVs directly from adipose tissue.

**Methods:** Characteristics and quantities of EVs were detected by transmission electron microscopy, nanoparticle tracking analysis, and Western blotting firstly. To investigate the biological effects of EVs on diabetic wound healing, angiogenesis, proliferation, migration, and inflammation-regulation assays were then evaluated *in vitro*, along with a diabetic wound healing mouse model *in vivo*. To further explore the potential therapeutic mechanism of EVs, miRNA expression profile of EVs were also identified and analyzed.

**Results:** The adipose tissue derived EVs (AT-EVs) were showed to qualify ISEV identification by nanoparticle tracking analysis and Western blotting and the AT-EVs yield from three methods was equal. EVs also showed promoting effects on biological processes related to diabetic wound healing, which depend on fibroblasts, keratinocytes, endothelial cells, and macrophages both *in vitro* and *in vivo*. We also observed enrichment of overlapping or unique miRNAs originate from different types of AT-EVs associated with diabetic wound healing for further investigation.

**Conclusion:** After comparative analyses, a mechanical method was proposed for preparing immediate clinical applicable EVs from adipose tissue that would result in reduced preparation time and lower cost, which could have promising application potential in treating diabetic wounds.

## Introduction

Treatment of diabetic wounds is a major challenge in clinical practice and places a heavy burden on both patients and medical systems (Olsson et al., 2019). As the population ages, the problem intensifies, as wound closure is negatively associated with age (Kloth, 2009; Wicke et al., 2009). Complications related to diabetic wounds include infection—such as cellulitis and infective venous eczema—gangrene, hemorrhage, and lower-extremity amputations, which lead to disabilities (Lin et al., 2019). Many therapeutic strategies have been widely used to attempt addressing the complex process of wound healing, such as the introduction of new dressings (Barnett and Varley, 1987; Mulder et al., 1993; Elliott, 2010; Miller et al., 2012) and skin substitutes (Hanft and Surprenant, 2002; Marston et al., 2003) local or systemic application of growth factors (Smiell et al., 1999; Emmerson et al., 2012), and physical interventions including hyperbaric oxygen (Löndahl et al., 2010) and negative pressure wound therapies (Masters et al., 2016). However, topical drugs have a short half-life, while biological products such as growth factors and skin substitutes, are limited by high cost and the modest single application effect (Han and Ceilley, 2017). Therefore, the development of more effective treatments remains a necessity.

In recent years, application of the mesenchymal stem cell (MSC)—an ideal therapeutic component that facilitates replacing “repair” with “regeneration”—has been reported to accelerate wound healing through enhancing re-epithelialization, increasing angiogenesis, modulating inflammation, and regulating extracellular matrix (ECM) remodeling (Yang et al., 2020). However, the use of MSCs is often accompanied by high risk of microvascular occlusion and unregulated growth during the treatment process (Wee Yeh Yeo et al., 2013). Recently, it has been reported that MSCs participate in tissue regeneration, predominantly by transferring information to damaged cells or tissue, through the release of extracellular vesicles (EVs) (Rani et al., 2015; Keshtkar et al., 2018), rather than directly differentiating into a specific cell type that promotes repair or repairs tissue damage. EVs are lipid bilayer-delimited particles with a diameter of 40–5,000 nm, which are secreted by cells or shed from cell membranes. They are widely found in various body fluids and cell supernatants, and offer stable loading of a variety of signaling molecules, including DNA, RNA and proteins, thereby playing a significant role in modulating intercellular communication (Li et al., 2021). Secretion-based MSC therapy has become an optimal treatment alternative, offering safer, cheaper, and more effective application in a variety of conditions. MSC-EVs reportedly fulfill multiple biological functions in the process of wound healing, such as promoting angiogenesis (Kang et al., 2016), facilitating immune regulation (Ha et al., 2020), and regulating cell proliferation and differentiation (Shabbir et al., 2015; Ren et al., 2019). Nevertheless, for the purpose of curing disease, large numbers of cells need to be cultured *in vitro*, to meet the abundant demand for EVs in clinical application (Gupta et al., 2021). This process is limited by the need for expensive equipment and reagents, extended time cost, and risk of contamination (Iannotta et al., 2021). Therefore, methods to extract EVs from discarded tissue need to be developed, for immediate application during surgery. Previous reports have shown that autologous fat grafting significantly promoted chronic or acute wound healing (Lolli et al., 2010; Sultan et al., 2012; Fettransplantation and Eine, 2014). This inspired our notion that adipose tissue may be a candidate for providing EVs to apply in diabetic wound healing, considering that large amounts thereof can be safely obtained in a minimally invasive manner, using liposuction (Zhang et al., 2017). Thus, we designed mechanical, collagenase-digestive, and constant *in-vitro*-collective methods for extracting adipose tissue-derived extracellular vesicles (AT-EVs). To evaluate the merits of each method, the quantities of EVs and EV-associated markers were identified, first. Thereafter, the EVs extracted using the different methods were applied in wound healing assays, both *in vitro* and *in vivo*, to evaluate their biological effects in diabetic wound healing and propose one method that most applicable for clinical treatment.

## Materials and methods

### Treatment of adipose tissue

**FIGURE 1:**
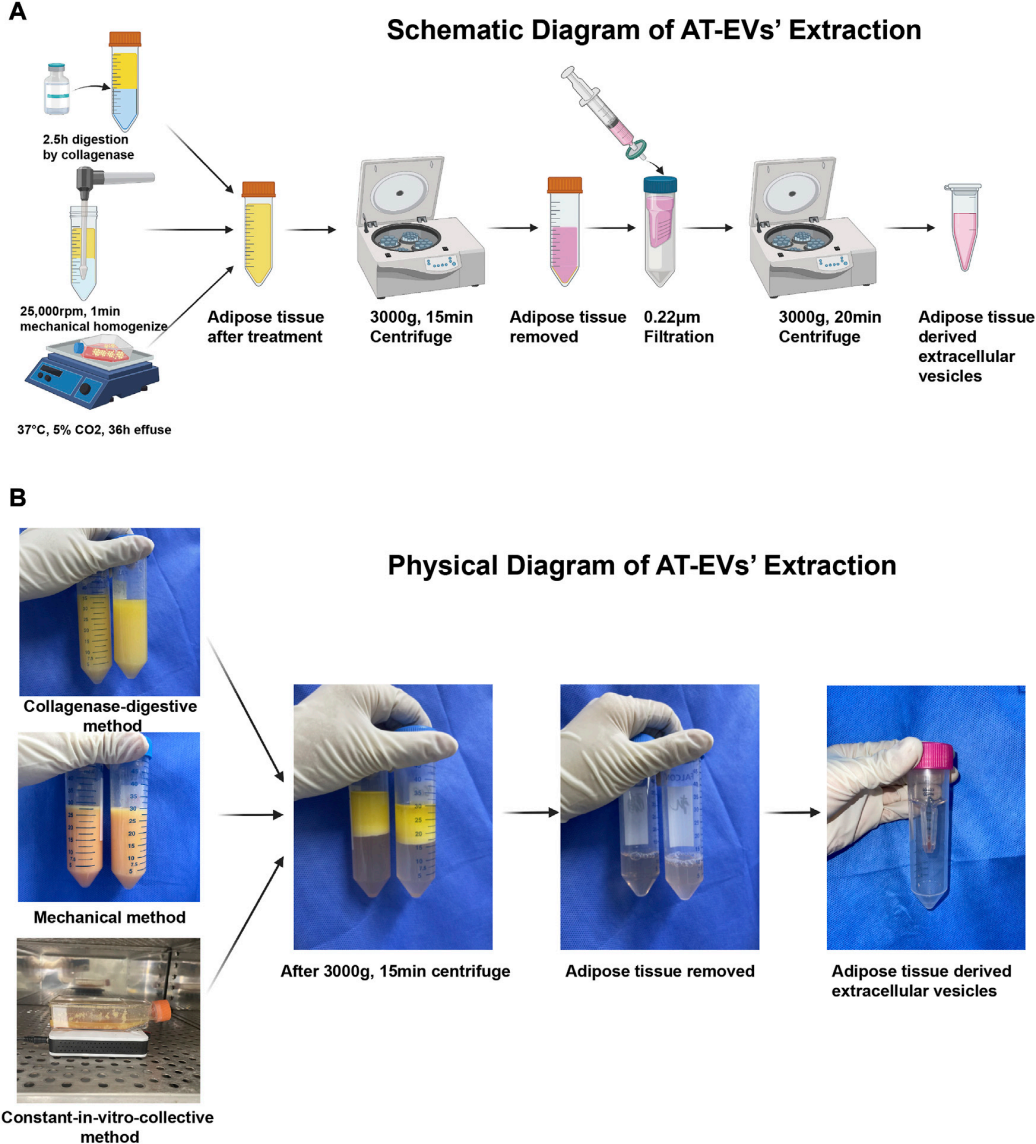
Isolation of adipose tissue-derived extracellular vesicles (AT-EVs) **(A)** Schematic diagram of the major steps involved in the extraction of AT-EVs **(B)** Physical diagram of the major steps involved in the extraction of AT-EVs.

Human adipose tissue was obtained from healthy female donors who had undergone abdominal liposuction. Written informed consent was provided by each patient and this study’s protocol was approved by the Ethics Committee of Shanghai Jiao Tong University School of Medicine. Adipose tissue was cleaned twice with saline, collected in a 50 mL centrifuge tube (BD Falcon #352070, Corning, Inc., Corning, NY, United States) and centrifuged at 1,500 rpm and 37°C for 5 min, to remove blood and tumescent fluid. In the collagenase-digestive method, 1,200 U commercial collagenase powder (QiaoYuan Biopharmaceutical, Shanghai, China) was dissolved in 30 mL phosphate-buffered saline (PBS, #20012, Gibco, NY, United States). The solution was added to 30 mL adipose tissue and the mixture incubated in a table concentrator at 37°C and 140 rpm, for 2.5 h (Figure 1A, top; Figure 1B, top). In the mechanical method, a mixture of 30 mL each of adipose tissue and PBS was mechanically homogenized for 1 min, using a handheld homogenizer at 25,000 rpm, until the adipose tissue was uniformly emulsified (Figure 1A, middle; Figure 1B, middle). In the constant *in-vitro*-collective method, adipose tissue was cultured with serum-free Eagle’s minimum essential medium, alpha-modified (α-MEM, #SH30265.01, HyClone, Logan, UT, United States) in a T25 culture bottle (#430639, Corning, Inc.) at 37°C, in a 5% CO_2_ incubator for 36 h. A magnetic stir bar was simultaneously introduced to the culture bottle and connected to a magnetic stirring apparatus to stir the mixture (Figure 1A, bottom; Figure 1B, bottom).

### Preparation of AT-EVs

After treatment of the adipose tissue, the mixture was collected and centrifuged at 4°C and 3,000 g for 15 min, followed by removal of all solid residue and retention of only the fluid supernatant (Figures 1A, B). EVs were extracted from the fluid using an ultrafiltration method as previously described (Chen et al., 2018; Liu et al., 2019; Xu et al., 2020). Briefly, the fluid was filtered through 0.22 μm filters (Millipore #SLGP033RS, Merck KGaA, Darmstadt, Germany) and concentrated using an Ultracel-100 membrane (100 kDa cut-off, Millipore #UFC910096, Merck KGaA) at 3,000 g and 4°C for 20 min, until the fluid content in the inner tube was less than 500 μL. The total AT-EV protein amount was determined using the bicinchoninic protein assay method according to the manufacturer’s protocol (BCA Protein Assay Kit, #P0012, Beyotime Biotechnology, Shanghai, China). After preparation, AT-EVs were stored at −80°C for the following applications: nanoparticle tracking analysis (NTA, Zeta View PMX 110, Particle Metrix, Meerbusch, Germany) and transmission electron microscopy (TEM, #HT7800, Hitachi, Tokyo, Japan). Western blotting was performed to identify AT-EVs. Henceforth, reference is made to dAT-EVs, mAT-EVs, and cAT-EVs, as derived using the collagenase-digestive, mechanical, and constant *in-vitro*-collective methods, respectively.

### Cell isolation and culture

Human immortal keratinocyte cells (HaCaTs), human umbilical vein endothelial cells (HUVECs), and mouse macrophage RAW 264.7 cells were purchased from the Cell Bank of the Chinese Academy of Science (Shanghai, China). Human dermal fibroblasts (HDFs) were isolated as previously described (Chen et al., 2007). Written informed consent was obtained from all patients and the study protocol approved by the Ethics Committee of Shanghai Jiao Tong University School of Medicine. All cells were cultured in high-glucose Dulbecco’s modified Eagle’s medium (DMEM, #C11995500BT, Gibco, Thermo Fisher Scientific, Inc., Bartlesville, OK, United States) supplemented with 10% fetal bovine serum (FBS, #16000044, Thermo Fisher Scientific, Inc.) and 1% antibiotic-antimycotic (#15240062, Thermo Fisher Scientific, Inc.). Cells were kept at 37°C in a 5% CO_2_ atmosphere incubator and sub-cultured upon reaching 80%–90% confluence, every 48–72 h. Concerning HDFs, cells of passage 2 were used for subsequent experiments.

### Western blotting

After total protein concentration was determined, AT-EVs—amounting to 30 μg protein—were loaded onto a 10%–15% sodium dodecyl-sulfate polyacrylamide gel electrophoresis (SDS-PAGE) gel and electrophoresed. AT-EV proteins were then transferred onto polyvinylidene difluoride (PVDF) membranes, incubated with primary anti-CD9 (1:1,000, #ab236630), anti-CD63 (1:1,000, #ab134045), anti-CD81 (1:1,000, #ab109201), anti-TSG101 (1:1,000, #ab125011), and anti-GM130 (1:1,000, #ab52649) antibodies (all obtained from Abcam, Cambridge, UK), followed by incubation with secondary antibodies (1:5,000, #111-035-045, Jackson ImmunoResearch, West Grove, PA, United States). Protein expression was evaluated using a BeyoECL Plus kit (#MA0186, Meilunbio, Dalian, Liaoning, China).

### Transmission electron microscopy

For TEM observation, dissociated fresh AT-EVs samples (20 μL) were loaded into a carbon-coated copper electron microscope grid for 5 min and negatively stained with a 5% uranyl acetate solution (SPI-02624, Head Biotechnology, Beijing, China) for 5 min. The mesh was then washed three times with PBS to remove the excess uranyl acetate solution and kept semi-dry using filter papers. The image was observed at 80 kV using a transmission electron microscope (#HT7800, Hitachi, Tokyo, Japan).

### Cellular uptake of AT-EVs

As previously described (Xu et al., 2020), 400 μg AT-EVs suspended in 500 μL PBS were labeled using 5 μL CellTracker CM-Dil Dye (#C7000, Invitrogen, Waltham, MA, United States of America) stock solution (1 mg/mL) and incubated at 37°C for 5 min, and 4°C for 15 min. After incubation, the CM-Dil labeled EVs were washed with PBS using ultrafiltration centrifugation three times at 3,000 g and 4°C for 10 min, as the molecular weight of CM-Dil (1.05 kDa) is far less than the weight cut-off of centrifugal filter Ultracel 100 (100 kDa). In this way, the unbound CM-Dil was removed. HDFs were incubated with CM-Dil-labeled AT-EVs for 12 h before being washed three times with PBS and fixed in 4% paraformaldehyde. After staining with phalloidin and 4′,6-diamidino-2-phenylindole (DAPI) (#40732ES10, Yeasen Biotechnology, Shanghai, China), cells were observed under a Nikon A1 confocal microscope (Nikon, Japan) to determine the uptake of labeled AT-EVs.

### Cell activity and proliferation assays

The cell activity of HUVECs/HDFs/HaCaTs was analyzed using a Cell Counting Kit-8 (CCK-8, #CK04-01, Dojindo Laboratories, Kumamoto, Japan), according to the provided instructions. After incubation with CCK-8 for 2 h, optical density (OD) values were detected using a microplate reader (Thermo Electron Corporation, Waltham, MA, United States), at a wavelength of 450 nm.

For the proliferation assay, HDFs/HaCaTs were co-cultured with 150 μg/mL AT-EVs for 2 days. Cell proliferation was evaluated using a 5-ethynyl-2′-deoxyuridine (EdU) DNA Cell Proliferation Kit (#C10310-1, Ruibo Biotechnology, Guangzhou, Guangdong, China) according to the manufacturer’s instructions. Briefly, cells cultured in a DMEM containing 10% FBS, 1% antibiotic-antimycotic, and 150 μg/mL AT-EVs in a 96-well plate, were incubated with EdU reagent for 2 h and fixed with formaldehyde, before being washed with glycine solution and PBS for 5 min, respectively. After being incubated with fluorescence dye Apollo and DAPI for 30 min, respectively, proliferating cells were observed under a fluorescence microscope (#Axio Vert. A1, Carl Zeiss, Jena, Germany). Five randomly selected images of each well were used to calculate the EdU-positive cell count, using ImageJ software 2.1.0. The rate of cell proliferation was calculated as follows:

$$Proliferation\;rate\;\left(\%\right)=\left(Number\;of\;EdU‐positive\;cells\right)\left(Total\;number\;of\;cells\right)\times 100\%$$

### Tube formation assay

Tube formation of HUVECs was evaluated as previously described (Xu et al., 2021). Briefly, 50 μL Matrigel (#356234, Corning Life Sciences, Bedford, MA, United States) was added to each well of a 96-well plate at 4°C and kept at 37°C for 30 min, to allow polymerization. HUVECs were seeded at a density of 7 × 10^3^ cells/well onto Matrigel and incubated with 100 μg/mL AT-EVs for 6 h. Tube formation was observed and imaged under an inverted light microscopy (Olympus, Tokyo, Japan). Five randomly selected fields of each well were used to calculate the total branch length and number of junctions, using ImageJ software 2.1.0.

### Scratch assay

The migration ability of HUVECs/HDFs/HaCaTs was evaluated by scratch assay, as previously described (Xu et al., 2021). Briefly, cells were cultured in six-well plates at a density of 4 × 10^5^ cells/well until reaching 100% confluence, followed by scraping across the plate with a 200 μL pipette tip and being washed thrice with PBS. Cells of the control group were cultured for 24 h in serum-free DMEM, whereas cells in other groups were co-cultured for 24 h in serum-free DMEM containing 100 or 150 μg/mL AT-EVs for HUVECs and HDFs/HaCaTs, respectively. Images were captured at 0 and 24 h using inverted microscopy (Olympus, Tokyo, Japan) and the area of the uncovered region was measured using ImageJ software 2.1.0. The rate of cell migration was calculated as follows:

$$Migration\;rate=\left(scratch\;area\;at\;0\;h-scratch\;area\;at\;24\;h\right)/scratch\;area\;at\;0\;h\times 100\%.$$

### Polarization induction and flow cytometry of RAW 264.7 macrophages

To investigate the role of AT-EVs in inflammation, 5 × 10^5^ RAW 264.7 macrophages were seeded in six-well plates and cultured with high-glucose DMEM with FBS, until cells reached 80% confluence. As previously described (Xu et al., 2020), cells were divided into five groups for which the culture medium was replaced as follows: **Control group**: cells were cultured with DMEM; **Lipopolysaccharide (LPS) + IFNγ group**: cells were cultured with DMEM +1 μg/mL LPS (#L2880, Sigma-Aldrich, St. Louis, MO, United States) + 30 ng/mL IFNγ (#315-05-100, PeproTech, Rocky Hill, NJ, United States); **LPS + IFNγ + dAT-EVs group**: cells were cultured with 1 μg/mL LPS +30 ng/mL IFNγ +150 μg/mL dAT-EVs; **LPS + IFNγ + mAT-EVs group**: cells were cultured with 1 μg/mL LPS +30 ng/mL IFNγ +150 μg/mL mAT-EVs; **LPS + IFNγ + cAT-EVs group**: cells were cultured with 1 μg/mL LPS +30 ng/mL IFNγ +150 μg/mL **c**AT-EVs. After induction for 24 h, 2 × 10^5^ cells were incubated with fluorescein isothiocyanate (FITC)-anti-mouse CD86 (1:50, #105110, BioLegend, San Diego, CA, United States) and PE-anti-mouse CD206 (1:40, #141706, BioLegend) at 4°C for 30 min, before being washed twice with staining buffer (#00-4222-26, Invitrogen, San Diego, CA, United States of America). Polarization of RAW 264.7 cells was determined using flow cytometry (BD FACSCalibur, Beckman Coulter, Inc., Brea, CA, United States).

### Quantitative real-time polymerase chain reaction

After RAW 264.7 macrophages were induced to polarize and co-cultured with AT-EVs for 24 h, total cellular mRNA was extracted using an EZ-press RNA Purification Kit (#B0004D, EZBioscience, Roseville, MN, United States) according to the manufacturer’s instructions. cDNA synthesis was then performed using a Reverse Transcription Master Mix (#A0010GQ, EZBioscience), followed by qPCR, using a SYBR green qPCR Master Mix (ROX2 plus, #A0001-R2, EZBioscience). The process settings were as follows: 5 min hot start at 95°C, 10 s at 95°C and 30 s at 60°C, for 40 cycles. Glyceraldehyde 3-phosphate dehydrogenase (GAPDH) was used to normalize gene expression levels. The relative mRNA expression levels were calculated using the 2^−ΔΔCT^ method, and the results expressed as fold-increases, relative to negative controls. qRT-PCR primers are shown in Table 1.

**TABLE 1:** Primers used for qRT-PCR.

Genes		Sequences (5′ to 3′)
Mouse GAPDH	F	CCC GTA GAC AAA ATG GTG AA
	R	TGC CGT GAG TGG AGT CAT AC
Mouse IL-6	F	CGG AGA GGA GAC TTC ACA GAG
	R	ATT TCC ACG ATT TCC CAG AG
Mouse TNF-α	F	CCA CTC TGA CCC CTT TAC TC
	R	GCC ATA ATC CCC TTT CTA AGT

### Immunofluorescence staining

For immunofluorescence staining of RAW 264.7 macrophages, 2 × 10^5^ cells were seeded in a 24-well plate and induced as described above, for 24 h, before being fixed in 4% paraformaldehyde for 10 min. Cells were then treated with 0.25% TritonX-100 (#9036-19-5, Sigma-Aldrich) for 10 min, according to the manufacturer’s instructions and washed three times with PBS, followed by blocking with FBS (#16000044, Thermo Fisher Scientific, Inc.) for 30 min at 25°C. After incubation with PE-conjugated anti-iNOS (1:500, #696806, BioLegend) at 4°C overnight, cells were stained using DAPI (#33342, Yansen Biotechnology, Shanghai, China) and observed under the fluorescence microscope (#Axio Vert. A1, Carl Zeiss). Five randomly selected images of each well were imaged (*n* = 3), and the number of iNOS-positive cells was calculated using the ImageJ software 2.1.0.

### Full-thickness cutaneous wound model

All animal experiments complied with the NIH’s Guide for the Care and Use of Laboratory Animals, with approval by the Animal Research Committee of Shanghai Jiao Tong University Affiliated Ninth People’s Hospital. A total of 40 male db/db mice (8 weeks old) were purchased from Gempharmatech (Shanghai, China). All mice were kept in a room with a 12 h light/dark cycle and stable temperature (25°C) and humidity. Mouse blood glucose levels and body weight were measured on days 0, 3, 7, 10, and 14 using a blood glucose meter (HGM-121, OMRON Healthcare Co., Ltd., Dalian, Liaoning, China) and an electronic scale (Hochoice Apparatus Manufacturer Co., Ltd., Shanghai, China), respectively.

After adapting to their environment for 1 week, mice were anesthetized by isoflurane inhalation, shaved, and each inflicted with an 8 mm, circular, full-thickness cutaneous wound (including the panniculus carnosus), by punch biopsy, as previously described (Yin et al., 2021). Hyaluronic acid (HA) hydrogel was used to deliver AT-EVs to create a moist environment for wound healing and fix AT-EVs *in situ*, as described in our previous publication (Liu et al., 2019). Prepared mice were randomly divided into five groups as follows: 1) **PBS group (control group)**: treated with 100 μL PBS; 2) **HA + PBS group**: treated with a mixture of 50 μL hyaluronic acid (HA, PureRegen^®^ Gel SINUS, BioRegen Biomedical Co., Ltd., Changzhou, Jiangsu, China) + 50 μL PBS; 3) **HA + dAT-EVs group**: treated with 50 μL HA + 50 μL dAT-EVs (400 μg); 4) **HA + mAT-EVs group**: treated with 50 μL HA + 50 μL mAT-EVs (400 μg); and 5) **HA + cAT-EVs group**: treated with 50 μL HA + 50 μL **c**AT-EVs (400 μg). All HA and fluid mixtures were directly applied to wound areas. Images of the wounds of all experimental mice were captured every 3 days, until the end of the experiment and analyzed using ImageJ software 2.1.0.

### Histology and immunofluorescence staining

All mice were euthanized after 14 days of treatment. Full-thickness, cross-sectional tissue from the wounded skin areas was harvested and fixed in 4% paraformaldehyde at 4°C overnight. After embedment in paraffin blocks, tissue was sectioned at 5 μm thickness for hematoxylin and eosin (H&E) and Masson’s trichrome staining to detect the formation of granulation tissue and collagen during wound healing.

For immunofluorescence staining, Ki67 immunofluorescence was used to evaluate the overall cell proliferation in the local wound area. CK14 immunofluorescence was used to evaluate keratinocyte differentiation and migration in the basal layer, and angiogenesis was evaluated using CD31 staining. As previously described (Xu et al., 2020), sections were conventionally dewaxed, hydrated, antigen-recovered, and blocked before incubation with primary anti-CK14 (1:100, #A19039, ABclonal Technology Co., Ltd., Wuhan, Hubei, China), anti-Ki67 (1:200, #ab16667, Abcam), anti-CD31 (1:1000, #ab182981, Abcam), or anti-CD68 (1:100, #sc-20060, Santa Cruz Biotechnology, Inc., Dallas, TA, United States) antibodies, and then incubated with FITC-conjugated (1:500, #111-095-003, Jackson ImmunoResearch) or PE-conjugated (1:500, #111-295-003, Jackson ImmunoResearch) secondary antibodies. Cell nuclei were stained using DAPI (1:1,000, #AR1176, Boster, Wuhan, Hubei, China). Five randomly selected fields of each specimen were imaged using a confocal microscope (#SP5, Leica, Wetzlar, Germany). The Ki67-and CD68-positive rates, as well as CD31-positive area were analyzed using ImageJ software 2.1.0. Positive rates were calculated as follows:

$$Positive\;rate\;\left(\%\right)=\left(Number\;of\;Ki67/CD68\;positive\;cells\right)/\left(Total\;number\;of\;cells\right)\times 100\%$$

### RNA extraction and library construction

Total RNA was extracted using a mirVana miRNA Isolation Kit (Ambion, Inc., Austin, TA, United States) according to the manufacturer’s protocol. Total RNA was quantified using the NanoDrop 2000 (Thermo Fisher Scientific, Inc.,). RNA integrity was assessed using an Agilent 2100 Bioanalyzer (Agilent Technologies, Inc., Santa Clara, CA, United States). The small RNA library was constructed using 1 μg total RNA from each sample and TruSeq Small RNA Sample Prep Kits (#RS-200-0012, Illumina, Inc., San Diego, CA, United States), following the manufacturer’s recommendations. Briefly, total RNA was ligated to adapters at each end, adapter-ligated RNA was reverse transcribed to cDNA, and PCR amplification was performed. PCR products ranging from 140 to 160 bp were isolated and purified as small RNA libraries. Library quality was assessed on the Agilent Bioanalyzer 2100 system, using DNA High Sensitivity Chips. The libraries were finally sequenced using the Illumina HiSeq X Ten platform. In total, 150 bp paired-end reads were generated. Small RNA sequencing and analysis were conducted by OE Biotech Co., Ltd. (Shanghai, China). The original data of small RNA sequencing was uploaded to the Sequence Read Archive (SRA) database (https://www.ncbi.nlm.nih.gov/sra/).

### Bioinformatics analysis

The basic reads were converted into sequence data (also called raw data/reads) by base calling. Low quality reads were filtered and those with 5′primer contaminants and poly (A) were removed. Reads without 3′adapter and insert tags, as well as those shorter than 15 nt or longer than 41 nt, were filtered from the raw data to obtain clean reads. For primary analysis, the length distribution of clean sequences in the reference genome was determined. Non-coding RNAs were annotated as rRNAs, tRNAs, small nuclear RNAs (snRNAs), and so forth. These RNAs were aligned and then subjected to the Bowtie (Langmead et al., 2009) search against Rfam v.10.1 (http://www.sanger.ac.uk/software/Rfam) (Griffiths-Jones et al., 2003). Known miRNAs were identified by alignment with the miRBase v22 database (http://www.mirbase.org/) (Griffiths-Jones et al., 2008), and known miRNA expression patterns in different samples were analyzed. Thereafter, unannotated reads were analyzed using miRDeep2 (Friedländer et al., 2012) to predict novel miRNAs. The corresponding miRNA star and mature sequences were also identified, based on the hairpin structure of the pre-miRNA and miRBase databases. Differentially expressed miRNAs were calculated and filtered against the threshold *p*-value <0.05. In turn, the *p*-value was calculated using the DEG algorithm (Anders, 2011) in the R package for experiments with biological replicates, along with the Audic-Claverie statistic (Tiňo, 2009) for experiments without biological replicates. The targets of differentially expressed miRNAs were predicted using the miRanda software (Enright et al., 2003) in animals, with the following parameters: S ≥ 150, ΔG ≤ −30 kcal/mol, and demanding strict 5′seed pairing. Gene Ontology (GO) and Kyoto Encyclopedia of Genes and Genomes (KEGG) pathway enrichment analyses of differentially expressed miRNA target genes were performed, respectively, using R based on the hypergeometric distribution.

### Statistical analysis

Data are reported as the mean ± standard deviation. Differences among groups were evaluated using one-way analysis of variance followed using Tukey’s posttest (Graph Pad Prism 9.1.1). Resultantly, 95% confidence intervals were calculated and differences deemed statistically significant at *p < 0.05.

## Results

### Preparation and characterization of AT-EVs

**FIGURE 2:**
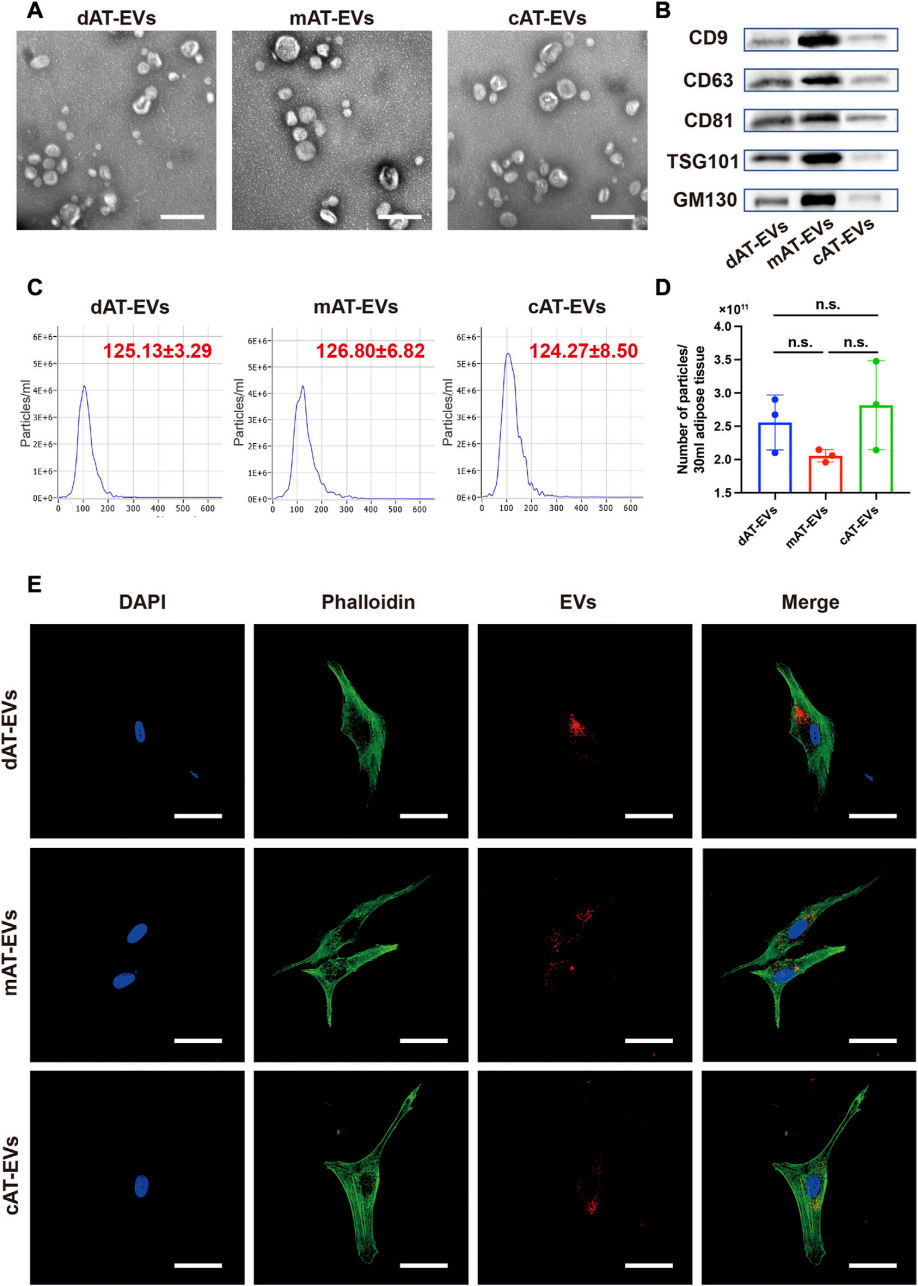
Characterization of AT-EVs **(A)** Morphology of AT-EVs, observed by transmission electron microscopy (TEM). Scale bars = 200 nm. **(B)** Expression of the EV-related markers (CD9, CD63, CD81, TSG101, and GM130) in AT-EVs, as determined by Western blotting. **(C)** Particle diameter distribution of dAT-EVs (125.13 ± 3.29 nm), mAT-EVs (126.80 ± 6.82 nm), and cAT-EVs (124.27 ± 8.50 nm), determined by nanoparticle tracking analysis (NTA). **(D)** Particle numbers of AT-EVs derived from 30 mL adipose tissue, measured using NTA. n = 3, n. s. no significant difference observed between groups **(E)** Immunofluorescence staining shows that AT-EVs were internalized by human dermal fibroblasts (HDF), after 12 h incubation. Scale bar = 50 μm.

After treatment of the adipose tissue, approximately two iterations of 30 mL PBS and 30 mL α-MEM containing EVs were collected, as derived from the collagenase-digestive, mechanical, and constant *in-vitro*-collective methods, respectively (Figure 1B). AT-EVs were extracted from α-MEM or PBS, using an ultrafiltration method. All EVs exhibited typical round or cup-shaped morphology (Figure 2A) with a diameter of 125.13 ± 3.29 nm (dAT-EVs), 126.80 ± 6.82 nm (mAT-EVs), and 124.27 ± 8.50 nm (**c**AT-EVs) (Figure 2C). Western blotting results showed that AT-EVs derived from all three methods, expressed EV-specific markers such as CD9, CD63, CD81, and TSG101, as well as the non-EV marker, GM130 (Figure 2B). The AT-EV yield from each of the collagenase-digestive, mechanical, and constant *in-vitro*-collective methods was (2.56 ± 0.41) × 10^11^ (2.06 ± 0.09) × 10^11^, and (2.82 ± 0.67) × 10^11^, respectively, without any significant difference (*p* > 0.05) (Figure 2D). A concentration of 1 mg/mL AT-EVs—determined by protein quantification—was equivalent to 1 × 10^11^ particles/mg, as determined by NTA. After incubation with HDFs for 12 h, we observed that AT-EVs from all three methods, had been internalized by the HDFs (Figure 2E), which demonstrated that all three methods successfully prepared AT-EVs from adipose tissue, without a significant difference in quantity.

### AT-EVs promoted cell activity and tube formation of HUVECs *in vitro*

**FIGURE 3:**
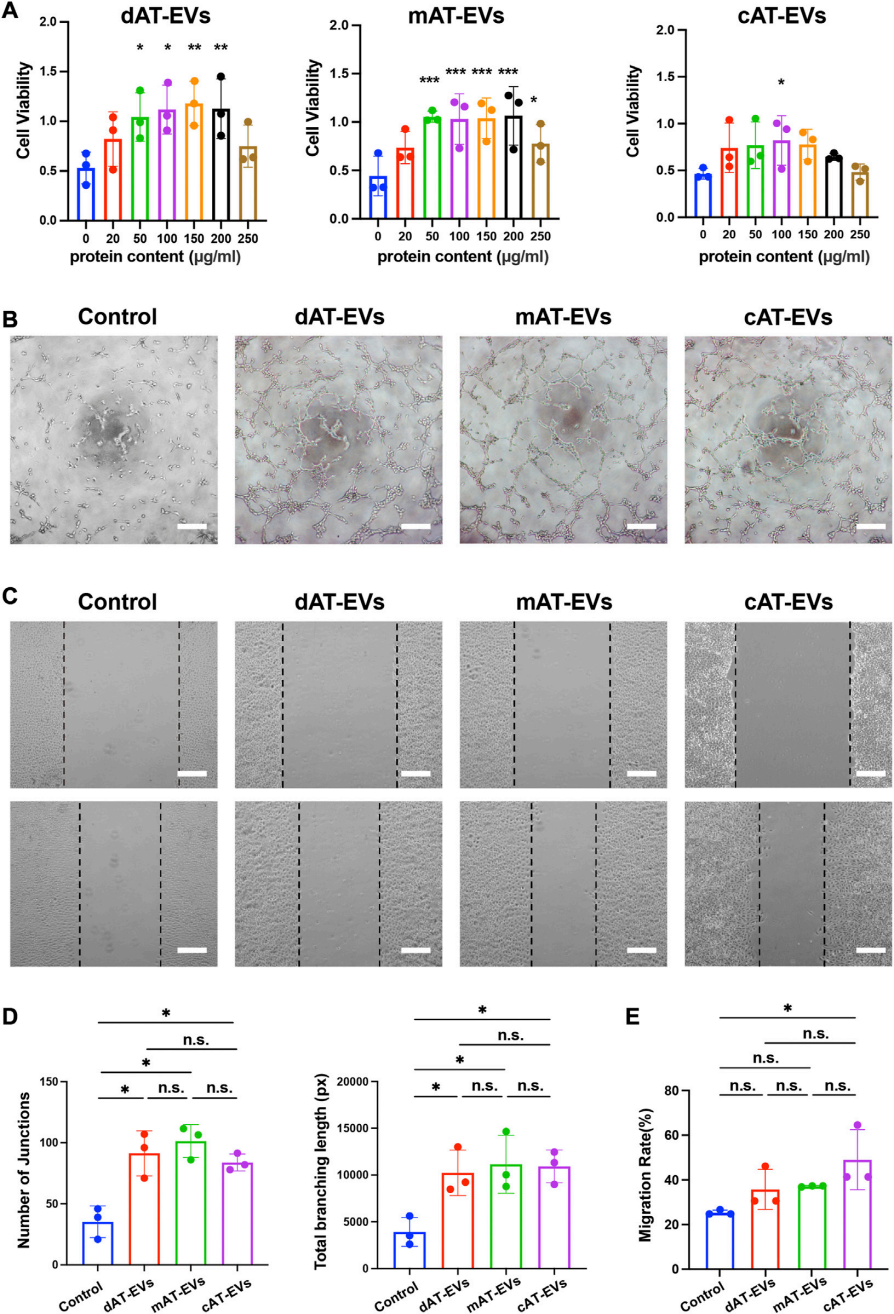
AT-EVs promoted the viability, tube formation, and migration of human umbilical vein endothelial cells (HUVECs) *in vitro* **(A)** Cell viability of HUVECs incubated with different concentrations of AT-EVs for 72 h (*n* = 3). **(B)** Tube formation of HUVECs, observed under a light microscope (*n* = 3). Scale bar = 250 μm. **(C)** Migration of HUVECs after 24 h incubation with 100 μg/mL AT-EVs (n = 3). Scale bar = 250 μm. **(D)** Statistical analysis of tube formation of HUVECs (total branching length and number of junctions) (*n* = 3). **(E)** Statistical analysis of HUVEC migration (*n* = 3). n. s. no significant difference observed between groups. *p < 0.05, **p < 0.01, ***p < 0.001.

To evaluate the effects of AT-EVs on HUVEC cell activity, PBS and gradient-based concentrations of the three AT-EV types, respectively, were incubated with HUVECs. After 72 h, CCK-8 results revealed significant (*p* < 0.05) cell viability-promoting effects at concentrations of 50–200 μg/mL of dAT-EVs and mAT-EVs, compared to that of the PBS group, whereas the same effects were observed only at 100 μg/mL of **c**AT-EVs. The highest concentrations (250 μg/mL) of dAT-EVs, mAT-EVs, and **c**AT-EVs showed somewhat inhibitory effects on cell viability, compared with the concentrations that promoted cell viability (Figure 3A). Therefore, based on its significant promotion of cell viability across all groups, 100 μg/mL was selected as the incubation dose for subsequent experiments.

After co-culture with PBS or 100 μg/mL AT-EVs for 6 h, tube formation assay results showed that tube formation was seldom observed in the PBS group. Contrastingly, obvious tube formation was observed in the dAT-EVs, mAT-EVs, and **c**AT-EVs groups (Figure 3B). Statistical analysis of the total branching length and number of junctions showed that all three AT-EV types significantly (*p* < 0.05) promoted tube formation of HUVECs, without significant (*p* > 0.05) difference among the groups (Figure 3D).

After incubation with PBS or 100 μg/mL AT-EVs for 24 h, migration assay results showed that, contrary to **c**AT-EVs, dAT-EVs and mAT-EVs exerted no significant (*p* > 0.05) migration-promoting effects on HUVECs (Figures 3C, E). These results demonstrated that dAT-EVs, mAT-EVs, and **c**AT-EVs were able to promote cell viability and tube formation of HUVECs, whereas only **c**AT-EVs promoted HUVEC migration.

### AT-EVs promoted cell activity, proliferation, and migration of HDFs *in vitro*

**FIGURE 4:**
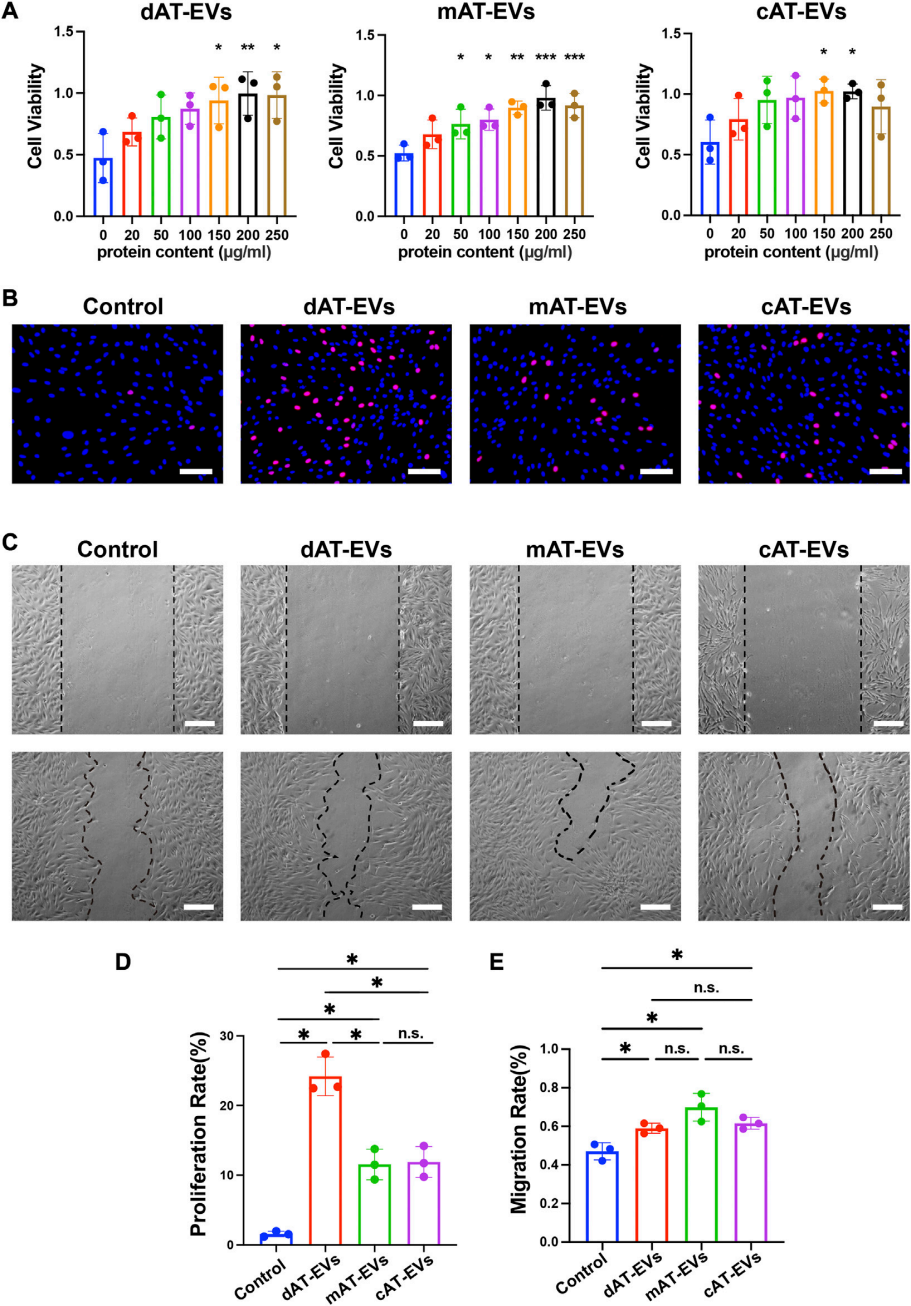
AT-EVs promoted cell activity, proliferation, and migration of HDFs *in vitro* **(A)** Cell viability of HDFs incubated with different concentrations of AT-EVs for 72 h (*n* = 3). **(B)** Cell proliferation determined using the EdU assay; EdU-positive HDFs (red) and nuclei (blue) were observed by fluorescence microscopy; scale bar = 100 μm. **(C)** Migration of HDFs after incubation with 150 μg/mL AT-EVs 24 h (n = 3). Scale bar = 250 μm. **(D)** Statistical analysis of HDFs’ proliferation rate (*n* = 3). **(E)** Statistical analysis of HDFs’ migration rate (*n* = 3). n. s. no significant difference observed between groups. *p < 0.05, **p < 0.01, ***p < 0.001.

To evaluate the effects of AT-EVs on HDF cell activity, PBS and gradient-based concentrations of different AT-EVs, respectively, were incubated with HDFs. After 72 h, CCK-8 results showed significant (*p* < 0.05) promotion of cell viability at concentrations of 150–250 μg/mL dAT-EVs, 50–250 μg/mL mAT-EVs, and 150–200 μg/mL **c**AT-EVs (Figure 4A). Following overall consideration, an AT-EV concentration of 150 μg/mL was selected for subsequent experiments on HDFs.

After incubation with PBS or 150 μg/mL AT-EVs for 48 h, EdU cell proliferation assay results showed a measure of cell proliferation in the control group. Cells treated with either dAT-EVs, mAT-EVs, or **c**AT-EVs exhibited significantly (*p* < 0.05) higher proliferation rates than those in the control group, without significant difference among the AT-EV groups (*p* > 0.05) (Figures 4B, D).

Following incubation with PBS or 150 μg/mL AT-EVs for 24 h, migration assay results showed that dAT-EVs, mAT-EVs, and **c**AT-EVs all significantly (*p* < 0.05) promoted HDF migration, compared with the PBS group, without significant difference among them (*p* > 0.05) (Figures 4C, E). These results demonstrated that dAT-EVs, mAT-EVs, and **c**AT-EVs were able to promote cell viability, proliferation, and migration of HDFs *in vitro*.

### AT-EVs promoted cell activity, proliferation, and migration of HaCaTs *in vitro*

**FIGURE 5:**
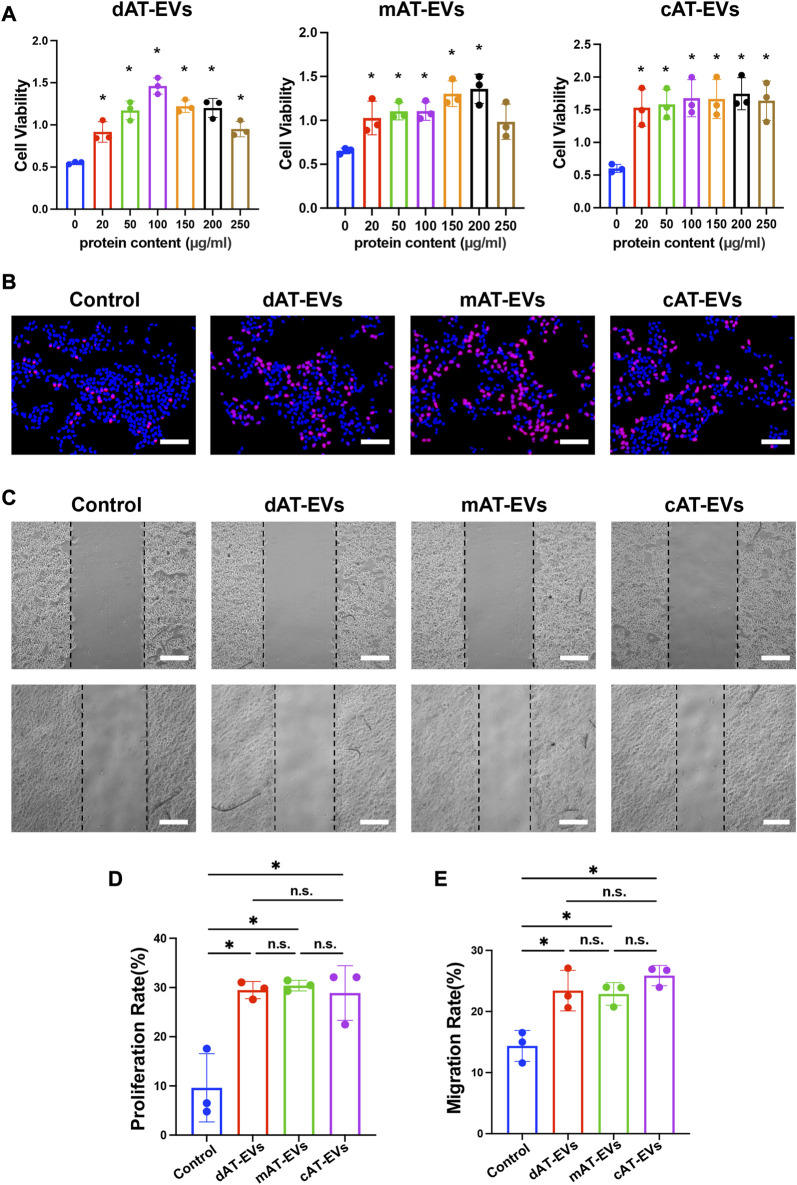
AT-EVs promoted cell activity, proliferation, and migration of HaCaTs *in vitro* **(A)** Cell viability of HaCaTs incubated with different concentrations of AT-EVs for 72 h (*n* = 3). **(B)** Cell proliferation determined using the EdU assay; EdU-positive HaCaTs (red) and nuclei (blue) were observed by fluorescence microscopy; scale bar = 100 μm. **(C)** Migration of HaCaTs after incubation with 150 μg/mL AT-EVs 24 h (*n* = 3). Scale bar = 250 μm. **(D)** Statistical analysis of HaCaTs’ proliferation rate (*n* = 3). **(E)** Statistical analysis of HaCaTs’ migration rate (*n* = 3). n. s. no significant difference observed between groups. *p < 0.05.

To evaluate the effects of AT-EVs on HaCaT cell activity, PBS and gradient-based concentrations of the different AT-EVs, respectively, were incubated with HaCaTs. After 72 h, CCK-8 results showed significant (*p* < 0.05) cell viability-promoting effects at concentrations of 20–250 μg/mL dAT-EVs, 20–200 μg/mL mAT-EVs, and 20–250 μg/mL cAT-EVs, compared to the PBS group (Figure 5A). Although these AT-EV concentrations promoted HaCaTs viability, compared to the control group, a trend was observed for high concentrations of AT-EVs to somewhat inhibit cell viability, compared to the medium concentrations. Following overall consideration, a concentration of 150 μg/mL AT-EVs was selected for subsequent experiments involving HaCaTs.

After being incubated with PBS or 150 μg/mL AT-EVs for 48 h, EdU cell proliferation assay results reflected a measure of cell proliferation in the control group. In comparison, dAT-EVs, mAT-EVs, and cAT-EVs all exhibited induction of a significantly (*p* < 0.05) higher proliferation rate than that observed in the control group, without significant difference among them (*p* > 0.05) (Figures 5B, D).

Following incubation with PBS or 150 μg/mL AT-EVs for 24 h, migration assay results showed that dAT-EVs, mAT-EVs, and cAT-EVs significantly (*p* < 0.05) promoted HaCaTs migration, without significant difference among the three groups (*p* > 0.05) (Figures 5C, E). These results demonstrated that dAT-EVs, mAT-EVs, and cAT-EVs were able to promote cell viability, proliferation, and migration of HaCaTs *in vitro*.

### AT-EVs decreased RAW 264.7 macrophages’ proinflammatory response

**FIGURE 6:**
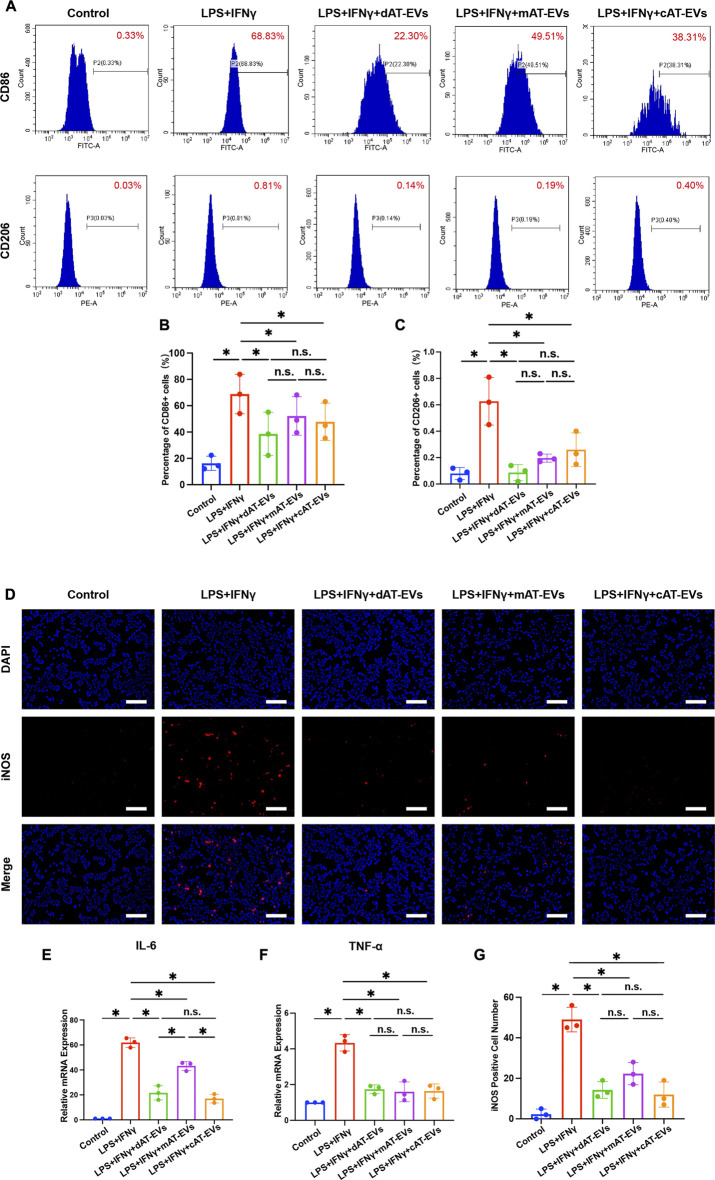
(Continued)

To investigate the role of AT-EVs in regulating the inflammatory response, RAW 264.7 cells were induced by proinflammatory factors and simultaneously incubated with 150 μg/mL AT-EVs for 24 h. Flow cytometry results indicated that LPS + IFNγ significantly induced RAW 264.7 cells from M0 (control group: 0.33% positive for CD86, an M1 macrophage marker) to M1 (LPS + IFNγ group: 68.83% positive for CD86) macrophages. All three types of AT-EVs exhibited a significant (*p* < 0.05) attenuating effect on the inflammatory response compared with that of the LPS + IFNγ group. However, no significant difference (*p* > 0.05) was observed among the three AT-EV types (Figures 6A, B). The CD206 (M2 macrophage marker) positive rate was below 1% in all groups, demonstrating that no M2 macrophages had been induced (Figure 6C). Accordingly, we focused on the effects of AT-EVs on M1 macrophage polarization, in subsequent experiments.

Immunofluorescence staining of iNOS (another M1 macrophage marker) showed that LPS + IFNγ significantly (*p* < 0.05) induced RAW 264.7 cells’ M1 polarization. Compared to the LPS + IFNγ group, dAT-EVs, mAT-EVs, and cAT-EVs all significantly (*p* < 0.05) decreased M1 polarization of RAW 264.7 cells, without significant (*p* > 0.05) difference among them (Figures 6D, G). qPCR results showed that LPS + IFNγ significantly (*p* < 0.05) induced expression of proinflammatory factors such as interleukin 6 (IL-6) and tumor necrosis factor α (TNF-α), whereas incubation with dAT-EVs, mAT-EVs, and cAT-EVs resulted in a diminished proinflammatory response. There was no significant (*p* > 0.05) difference among the three types of AT-EVs in terms of TNF-α expression, but significantly decreased expression of IL-6 was observed in the LPS + IFNγ + dAT-EVs and LPS + IFNγ + cAT-EVs groups compared with that in the LPS + IFNγ + mAT-EVs group (Figures 6E, F). These results demonstrated that dAT-EVs, mAT-EVs, and cAT-EVs all exhibited the ability to attenuate the proinflammatory response.

### AT-EVs promoted skin wound healing in diabetic mice

Both the blood glucose levels and body weight of db/db mice were evaluated during the wound healing process. All diabetic mice in each group exhibited typical blood glucose levels exceeding 20 mmol/L and body weight greater than 40 g. There were no significant (*p* > 0.05) differences in blood glucose levels and body weight between the PBS and all treatment groups (Supplementary Figure S1), indicating the same parameter baseline in all mouse groups.

**FIGURE 7:**
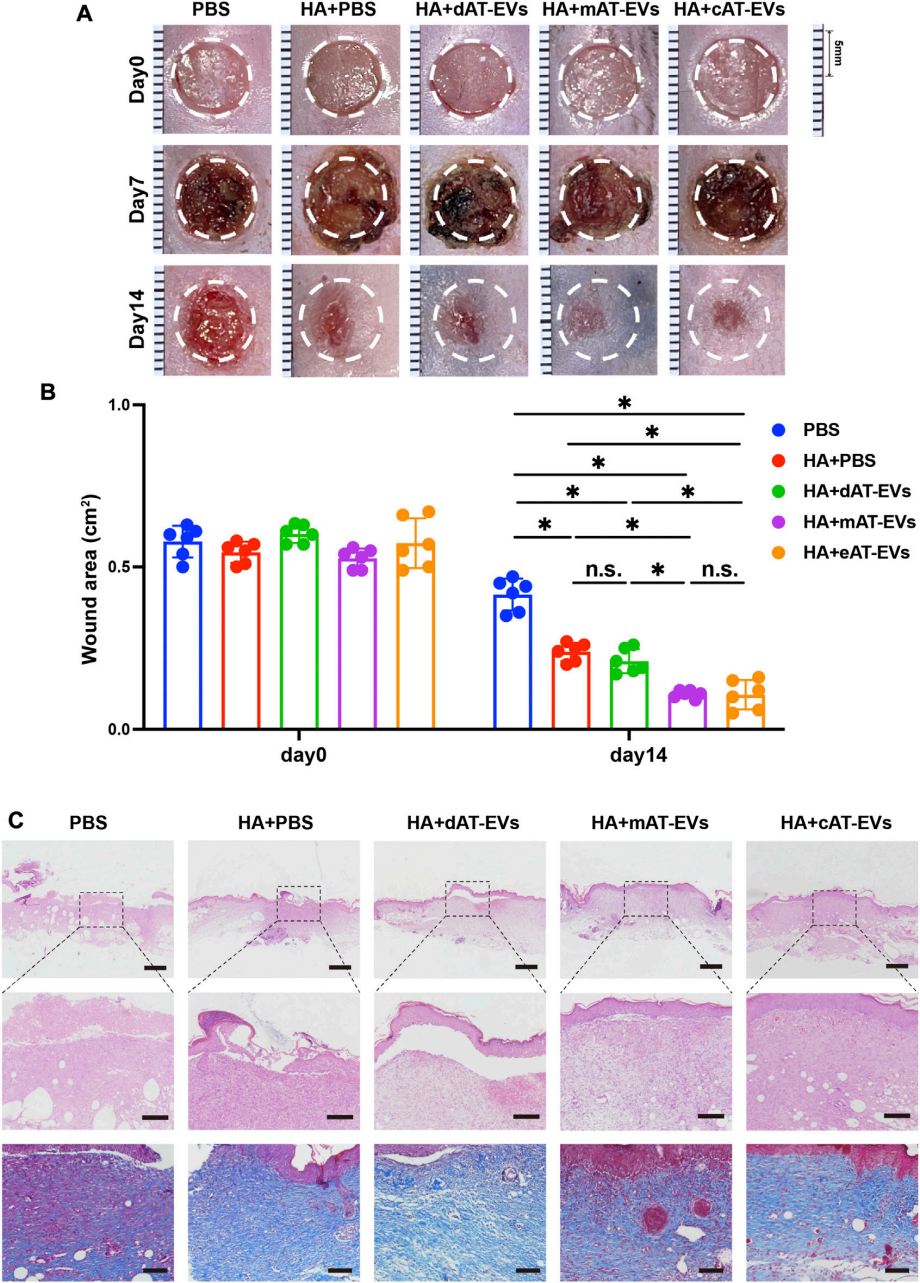
AT-EVs promoted chronic wound healing in diabetic mice **(A)** Representative photographs of mouse wounds at days 0, 7, and 14. Scale bar = 1 mm. **(B)** Statistical analysis of the wound area (*n* = 6). **(C)** H&E and Masson staining of wound tissue sections, observed under a light microscope. Scale bar = 500 μm (top), 150 μm (middle), 100 μm (bottom).

Upon gross observation, no obvious contraction, infection, or necrosis were observed in any mice, during the wound healing process. On day 7, dark brown blood scabs and granulation tissue were observed on the skin wound areas. Statistical analysis was not shown as the actual wound area could not be accurately measured. However, by day 14, the wound areas of the HA + PBS, HA + dAT-EVs, HA + mAT-EVs, and HA + cAT-EVs groups had decreased significantly (*p* < 0.05) compared with those of the PBS group. Moreover, the HA + mAT-EVs and HA + cAT-EVs groups showed significantly (*p* < 0.05) smaller wound areas than the HA + PBS group, without significant (*p* > 0.05) difference between the two AT-EV groups. In contrast, the wound areas of mice in the HA + dAT-EVs group were statistically equal to those of mice in the HA + PBS group (Figures 7A, B).

Upon microscopic observation on day 14, H&E staining results revealed a large skin defect, spanning epidermal and dermal tissue, in the PBS group, with some blood scab covering. Compared with the PBS group, the HA + PBS group showed certain decreased defects with fewer epidermal and dermal defects. Moreover, a cracked epidermis and intact dermis were observed in the HA + dAT-EVs group, whereas nearly intact epidermis and dermis were observed in the HA + mAT-EVs and HA + cAT-EVs groups. Masson’s trichrome staining results showed broken and disorganized collagen fibers in the PBS group but more abundant and organized collagen fibers in the HA + PBS and HA + dAT-EVs groups. Both increased deposition and a more oriented arrangement of collagen fibers were observed in the HA + mAT-EVs and HA + cAT-EVs groups compared to the HA + PBS and HA + dAT-EVs groups (Figure 7C). These results suggested that AT-EVs—mAT-EVs and cAT-EVs, in particular—were able to promote diabetic wound healing by re-epithelization with collagen deposition and rearrangement *in vivo*.

### AT-EVs promoted epidermis formation, cell proliferation, and angiogenesis and attenuated inflammation *in vivo*

**FIGURE 8:**
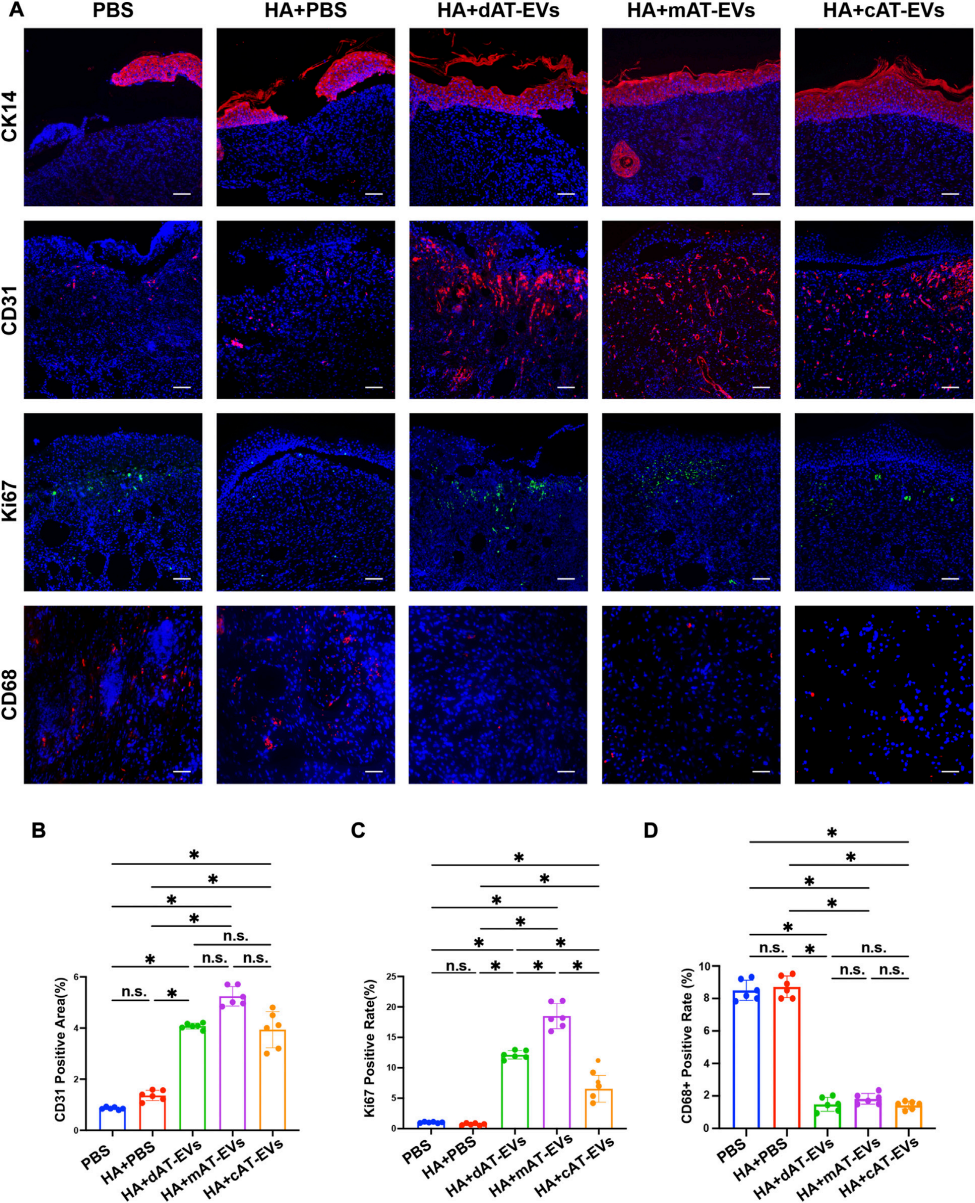
AT-EVs promoted cell proliferation, keratinocyte differentiation and migration, and angiogenesis while decreasing inflammation *in vivo* **(A)** Immunofluorescent staining of CK14 (red), CD31 (red), and Ki67 (green), representing keratinocyte differentiation and migration, angiogenesis, and cell proliferation, respectively, as well as CD68 (red), representing inflammatory cells, observed under a fluorescence microscope. Scale bar = 75 μm. **(B)** Statistical analysis of CD31-positive area (*n* = 6). **(C)** Statistical analysis of Ki67-positive cells (*n* = 6). **(D)** Statistical analysis of CD68-positive cells (*n* = 6).

To further explore the mechanism by which AT-EVs promote diabetic wound healing, the formation of new epidermis, along with angiogenesis and cell proliferation, and inflammatory response were detected using immunofluorescence staining of markers CK14, CD31, Ki67, and CD68, respectively. CK14 staining results illustrated that the mAT-EVs and cAT-EVs groups presented with newly generated epidermis that was complete, thicker, and compact. In contrast, the epidermis of the PBS, HA + PBS, and HA + dAT-EVs groups was rather thin or interrupted, indicating that mAT-EVs and cAT-EVs had more pronounced effects on promoting formation of new epidermis (Figure 8A). Meanwhile, CD31 staining results revealed a significantly (*p* < 0.05) larger angiogenesis area in the HA + dAT-EVs, HA + mAT-EVs, and HA + cAT-EVs groups compared with the PBS and HA + PBS groups, without significant difference (*p* > 0.05) among the AT-EV groups. There was also no significant difference (*p* > 0.05) between the PBS and HA + PBS groups, in terms of the angiogenesis area (Figures 8A, B). Moreover, Ki67 staining results showed that cell proliferation was not significantly (*p* > 0.05) increased in the HA + PBS group compared with the PBS group, whereas it was significantly increased in the HA + dAT-EVs, HA + mAT-EVs, and HA + cAT-EVs groups. Compared with the HA + PBS group, cell proliferation was also significantly (*p* < 0.05) increased in the HA + dAT-EVs, HA + mAT-EVs, and HA + cAT-EVs groups. Furthermore, cell proliferation in the HA + mAT-EVs group was significantly (*p* < 0.05) higher than that in the HA + dAT-EVs group, which was, in turn, significantly (*p* < 0.05) higher than that in the HA + cAT-EVs group (Figures 8A, C). CD68 staining results revealed no significant (*p* > 0.05) difference in macrophage infiltration, between the PBS and HA + PBS groups. In comparison, significantly (*p* < 0.05) decreased macrophage infiltration was observed in the HA + dAT-EVs, HA + mAT-EVs, and HA + cAT-EVs groups, without significant (*p* > 0.05) difference among them (Figures 8A, D). These results demonstrated that AT-EVs were able to promote new epidermis formation, angiogenesis, and cell proliferation, as well as attenuate the inflammatory response in the diabetic wound healing process.

### Expression profiling of AT-EV miRNAs

**FIGURE 9:**
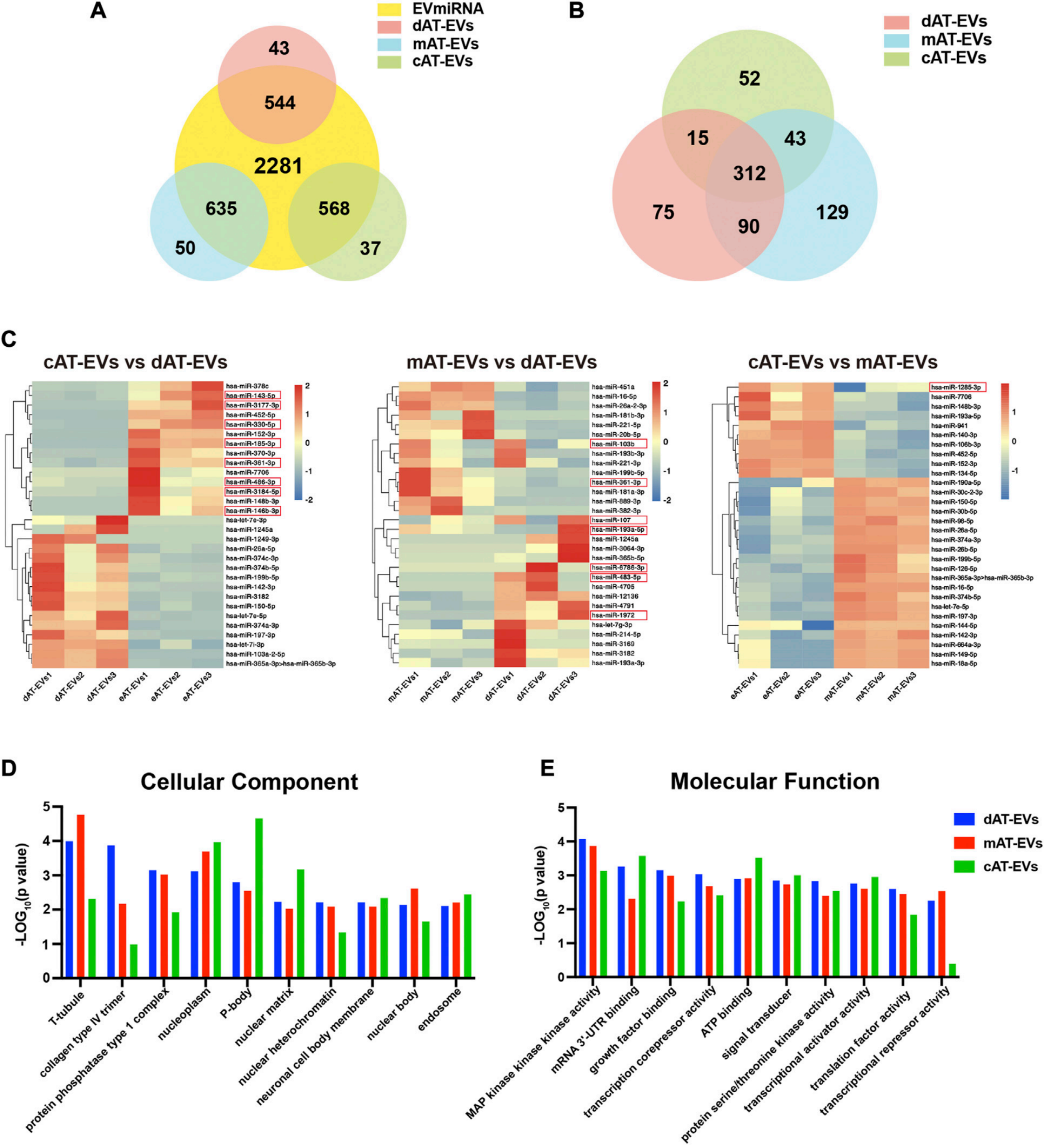
Bioinformatics analysis of miRNA enriched in AT-EVs **(A)** Venn diagram illustrating the number of miRNAs in dAT-EVs, mAT-EVs, and cAT-EVs, respectively, overlapping with those in the EVmiRNA database. **(B)** Venn diagram illustrating the number of overlapping miRNAs between dAT-EVs, mAT-EVs, and cAT-EVs **(C)** Hierarchical clustering of differentially expressed miRNAs (fold change >2.0 and *p* < 0.01; unreported miRNAs, as well as those with low TPM values, were omitted) between cAT-EVs and dAT-EVs (left), mAT-EVs and dAT-EVs (middle), and cAT-EVs and mAT-EVs (right), respectively. Rows represent miRNAs, whereas columns represent individual replicates. **(D–E)** Gene ontology (GO) analysis of predicted differentially expressed genes of cellular components and molecular functions, based on the identified differentially expressed miRNAs.

Based on the biological functions of AT-EVs *in vivo* and *in vitro*, we further identified and analyzed the miRNA expression profile of AT-EVs, to explore their potential therapeutic mechanism. The miRNA sequences of dAT-EVs, mAT-EVs, and cAT-EVs were compared with the miRNA recorded in the EVmiRNA database (http://bioinfo.life.hust.edu.cn/EVmiRNA); overlap rates were 544/587, 635/685, and 568/605, respectively, further supporting the EV-related origin of the miRNAs (Figure 9A). In total, 312 common miRNAs were detected among the three types of AT-EVs, along with additional unique miRNAs. The differential expression is displayed in a cluster heatmap (Figures 9B, C). The top-30 miRNAs identified from the pairwise comparison of the three AT-EV types, according to ascending arrangement of *p-*values, were included in the cluster heatmap. The names of miRNAs were searched in Pubmed (https://pubmed.ncbi.nlm.nih.gov/), and the relevant miRNAs associated with wound healing—typically miR-107, miR-193a-5p, miR-6786-3p, miR-483-5p, and miR-1972 in dAT-EVs; miR-103b and miR-361-3p in mAT-EVs; and miR-143-5p, miR-3177-3p, miR-330-5p, miR-185-3p, miR-361-3p, miR-486-3p, miR-3184-5p, miR-146b-3p, and miR-1285-3p in cAT-EVs (Figure 9C) — were manually selected according to previous reports and highlighted.

**FIGURE 10:**
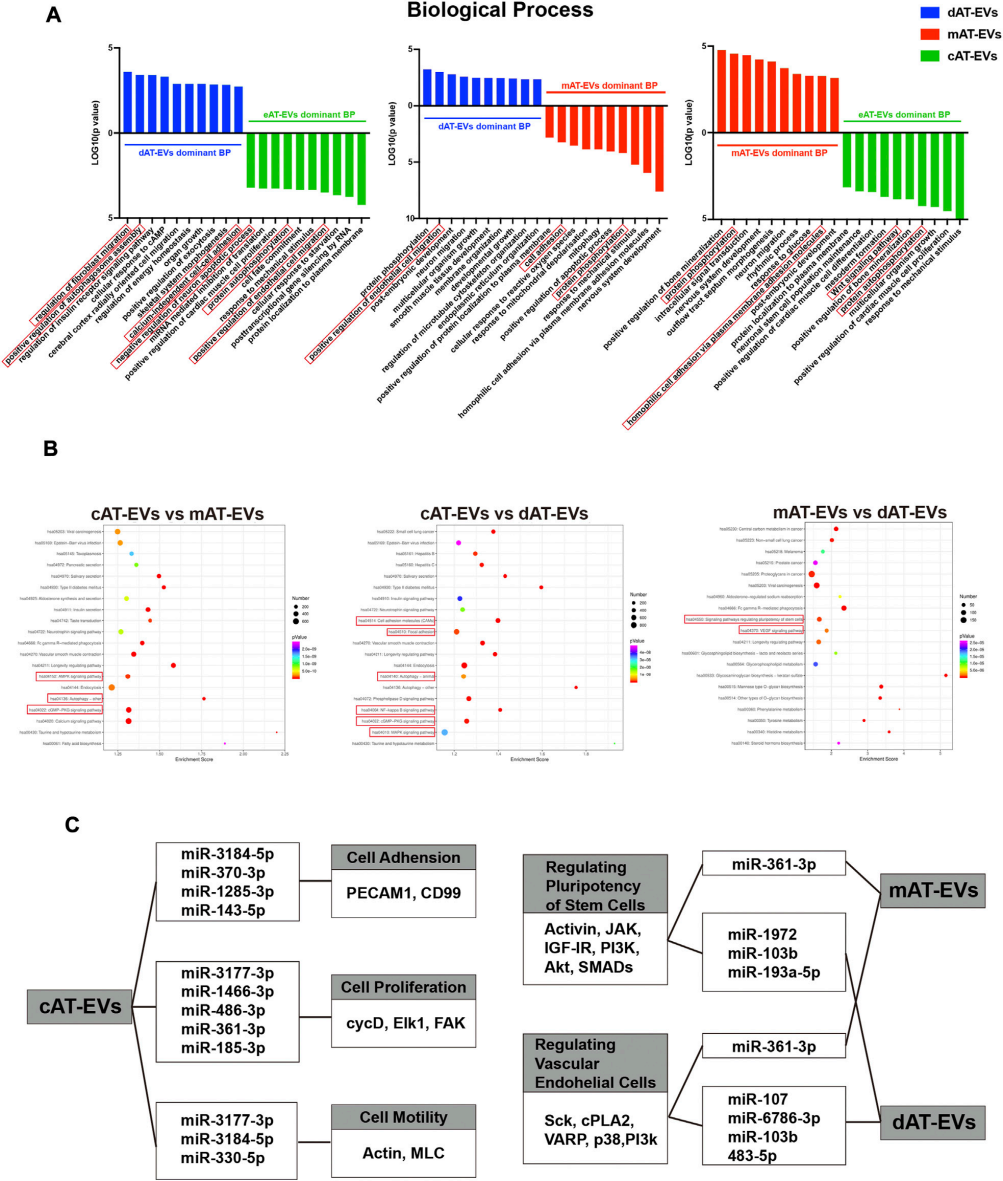
Gene Ontology (GO) and Kyoto Encyclopedia of Genes and Genomes (KEGG) analyses of miRNA in AT-EVs **(A)** GO analysis of predicted differentially expressed genes of biological processes, based on the identified differentially expressed miRNAs. **(B)** KEGG pathway analysis of differentially expressed genes. The 20 most enriched terms are presented as bubble charts and the wound healing-associated pathways, highlighted **(C)** Enriched functions and pathways associated with wound healing, with their corresponding miRNAs and differentially expressed genes. TPM: transcripts per million.

The top 10 GO terms of cellular components (CC) of the three types of AT-EVs were selected, according to miRNA expression. The results showed that dAT-EVs contained miRNAs closely related to T-tubule and collagen type IV trimer, mAT-EVs contained miRNAs closely related to T-tubule and nucleoplasm, whereas cAT-EVs contained miRNAs closely related to nucleoplasm and p-body, indicating that the three AT-EVs were derived from similar cellular components but also varied from one another in certain aspects (Figure 9D). The predicted molecular functions, including various bindings, transcription factor activities, and enzymatic activities, were similar among the three AT-EV types (Figure 9E). The dominant biological processes of dAT-EVs included regulation of fibroblast migration, positive regulation of autophagosome assembly, and calcium-independent cell-cell adhesion. mAT-EVs were mostly involved in positive regulation of endothelial cell migration, cell adhesion, protein phosphorylation, and homophilic cell adhesion *via* plasma membrane adhesion molecules, whereas cAT-EVs were largely responsible for negative regulation of neuron apoptotic processes, protein autophosphorylation, and positive regulation of endothelial cell migration (Figure 10A). Considering the greater promotion of skin wound healing facilitated by the HA + mAT-EVs and HA + cAT-EVs groups, compared with that of the HA + dAT-EVs group, KEGG analysis further confirmed the enrichment of pathways related to wound healing in cAT-EVs and mAT-EVs. These pathways included autophagy, cell adhesion molecules, and focal adhesion, as well as the AMPK (an inflammation regulating signaling pathway (Qing et al., 2019), cGMP-PKG (an angiogenesis and keratinocyte cell migration regulating signaling pathway (Siamwala et al., 2010; Zhan et al., 2015), NF-kappa B (a signaling pathway associated with wound healing (Park et al., 2018)), MAPK (an angiogenesis-related signaling pathway (Tao et al., 2017), and VEGF (another angiogenesis-related signaling pathway (Zhu et al., 2019), along with the pathway regulating pluripotency of stem cells (Figure 10B). The differentially expressed miRNAs of AT-EVs and their target genes that are closely related to wound healing, as predicted using KEGG analysis, are shown in Figure 10C. In summary, miRNA sequencing results showed enrichment of miRNAs related to skin wound healing—which regulate skin wound healing *via* multiple processes—in all three types of AT-EVs.

## Discussion

In this study, we proposed three methods—mechanical, collagenase-digestive, and constant *in-vitro*-collective—to rapidly prepare EVs from adipose tissue, for immediate clinical application. No significant differences in morphology, particle number, vesicle diameter, or uniformly expressed EV-related surface markers (CD9, CD63, CD81, TSG101) (Théry et al., 2018), were observed among the AT-EVs extracted *via* the three methods. Moreover, all AT-EV types could be internalized by HDFs, indicating effective preparation and enrichment of EVs from adipose tissue. In addition, all three types of AT-EVs promoted angiogenesis, proliferation, and migration of fibroblasts and epidermal cells, and reduced M0-to-M1 polarization of inflammatory cells *in vitro*. Moreover, mAT-EVs and cAT-EVs significantly accelerated diabetic wound healing in db/db mice *in vivo*.

Considering that EVs generally play an important role in regulating intercellular communication through their secretion by cells, they mainly exist in the intercellular substance (Cheng and Hill, 2022). Therefore, in order to dissociate adipose tissue and expose the intercellular space, we first designed collagenase-digestive and mechanical methods, respectively, to dissociate adipose tissue, which was inspired by the classic extraction method of primary cells (Estes et al., 2010). Furthermore, aspirated granular fat was immersed in α-MEM and continuously stirred, to collect the EVs constantly released by cells in adipose tissue into the fluid, according to previous reports (Gesmundo et al., 2021; Hong et al., 2021). After dissociation and centrifugation of adipose tissue, the low-density components—including oil and adipose cells—floated in the upper layer while cell fragments collected in the lowest layer, so that majority of the EVs were theoretically enriched in the intermediate fluid layer. Upon completion of the above process, we successfully prepared and concentrated EVs from the intermediate fluid layer, using an ultrafiltration method. In this study, the collagenase-digestive method used commercial collagenase, which is routinely used in the clinical treatment of intervertebral disc protrusion and can be directly applied in our practice. This preparation process requires availability of a 37°C shaking table combined with the centrifuge aforementioned and takes 4 h to prepare AT-EVs. Furthermore, the constant *in-vitro*-collective method requires availability of a good manufacturing practice (GMP) laboratory and takes nearly 40 h for AT-EV preparation, but its process does not necessitate the addition of serum or other organic substances; it is thus safer and faster than the method of collecting supernatant from cultured cells. Finally, our proposed mechanical method—which requires 1 h for AT-EV extraction—only calls for availability of a sterilized mechanical homogenizer, combined with an ordinary centrifuge that can reach 3,000 g*,* fully fit our goal for instant preparation of EVs during surgery, which can be applied in clinical treatment and popularized among surgeons.

In this study, we selected liposuction to acquire adipose tissue, which has been used for decades in plastic surgery with a very low incidence of major complications (Cárdenas-Camarena et al., 2011). The use of autologous adipose tissue in the treatments of skin ulcers in patients with diabetes or critical limb ischemia has been reported in several clinical trials, demonstrating that microvascular injuries and other complications associated with liposuction are limited, even in patients with diabetes (Lee et al., 2012; Lonardi et al., 2019; Armstrong et al., 2022). However, liposuction surgery may still be a challenge for patients who suffer from drastic systemic effects of diabetes, and the application of allogeneic AT-EVs is still a major interest for future research.

The results of NTA showed that there was no statistical difference in the number of EVs obtained from the three different methods, using the same volume of adipose tissue. This indicated that, compared with the other two methods, the mechanical method—which only consumes 1 h for EV preparation—was more suitable for rapid extraction in a clinical setting. Although Western blot identification showed that all prepared EVs, regardless of the method used, expressed EV-specific markers such as CD9, CD81, CD63, and TSG101, the non-EV marker was simultaneously positively expressed (Figure 1B). This demonstrated that EVs prepared by the three methods represented combinations of EVs and non-EV components, and their specific composition requires further identification. It is also possible that the mechanical process of opening cells and intercellular spaces to capture EVs also chopped the cells into EV-mimetics using shear forces (Du et al., 2022), which may also explain why mAT-EVs exhibited highest GM130 expression. The contamination with non-EV material may also be attributed to usage of the ultrafiltration method. However, from the point of view of speedy preparation and application during an operation, the ultrafiltration method can be applied to quickly derive sufficient EVs as compared with density gradient ultracentrifugation (this method has been reported as the gold standard for obtaining the purest EVs, but it is extremely time-consuming and involves tedious steps, leading to limitation in clinical use) (Lobb et al., 2015; Seo et al., 2022). In this study, the mechanical method met the requirement of being clinically applicable. In addition, immunofluorescence staining showed that AT-EVs could be internalized by HDFs. A single cell view was shown to distinguish the details of EVs internalized by HDFs as the EV sizes were too small to be observed under a larger viewing field, limiting analysis of the percentage of cells that internalized EVs.

Considering the mixed nature of the AT-EVs derived in this study, their efficiency in promoting diabetic skin wound healing needed to be fully evaluated *in vitro* and *in vivo*. Wound healing is a complex process, which involves a complicated interplay between angiogenesis (Eming et al., 2007), proliferation and migration of epidermal (Xia et al., 1999) and dermal cells (Regan et al., 1991), and an inflammatory reaction, both in the early and late stages of repair (Sindrilaru et al., 2011). Based on this theoretical foundation, we first verified whether the three AT-EV types could promote angiogenesis, accelerate the proliferation and migration of epidermal and dermal cells, and regulate inflammation *in vitro*, which were validated using HUVECs, HaCaTs, HDFs, and RAW 264.7 macrophages, respectively. In identifying the effects of AT-EVs on HUVECs, the three AT-EV types promoted angiogenesis equally, while only cAT-EVs could significantly enhance cell migration. However, no difference in angiogenesis was observed upon CD31 staining *in vivo*, indicating that AT-EVs may have mainly promoted angiogenesis through a direct re-vascularization effect, rather than promoting endothelial cell migration. Moreover, all three AT-EVs showed promotion of cell viability at relatively lower concentrations, with a decreased promoting effect at higher concentrations, similar to that of resveratrol on breast cancer cell (Nakagawa et al., 2001). The reduction of cell viability at higher EVs concentrations was possibly due to increasing autophagy or apoptosis, according to research on resveratrol and breast cancer cells (Cheng et al., 2022). Furthermore, the three AT-EV types equally promoted the migration of HDFs and HaCaTs, as well as the proliferation of HaCaTs, while dAT-EVs significantly promoted HDF proliferation compared with that of mAT-EVs and cAT-EVs. However, the results of *in vivo* Ki67 staining illustrated that the HA + mAT-EVs group exerted a stronger cell proliferation effect than the dAT-EVs group. This may be attributed to mAT-EVs promoting the proliferation of wound healing-associated skin cells other than HDFs and HaCaTs, such as hair follicle stem cells and peripheral neurons (Rajendran et al., 2017; Carrasco et al., 2019; Ma et al., 2019; Cha et al., 2020). However, these effects would require further investigation in the future. The effects of AT-EVs on angiogenesis, proliferation, and migration of epidermal and dermal cells were similar to and comparable with those described in previous publications (Shabbir et al., 2015; Zhang et al., 2015; Li et al., 2016; Guo et al., 2017; Yang et al., 2020) on EVs derived from other cells or plasma sources such as umbilical cord stem cells, human endothelial progenitor cells, and platelet rich plasma.

In addition, inflammation plays an important role in wound healing, from the immediate moment of injury through the late remodeling stage (Broughton et al., 2006; Baltzis et al., 2014). Excessive inflammatory reactions—especially during the late stage of wound healing—have been reported by many studies on delayed diabetic wound healing (Pradhan Nabzdyk et al., 2013; Tellechea et al., 2013). Similar to previous studies regarding ADSC-EVs’ or BMSC-EVs’ attenuation of the inflammatory reaction (He et al., 2019; Xu et al., 2020), our results demonstrated that the three types of AT-EVs could nearly equally inhibit the inflammatory response, by reducing the polarization of macrophages from M0 to M1. *In vivo* CD68 staining conformed to the *in vitro* findings that AT-EVs could decrease macrophage infiltration. These phenomena coincided with those observed in granular fat grafting for inflammatory diseases (Premaratne et al., 2011; Bouglé et al., 2018; Iyengar et al., 2019). The anti-inflammatory response effects may be traced to the adipose-derived stem cells (ADSCs), as well as intrinsic T cells and macrophages in adipose tissue, considering the characteristics of the adipose-tissue origin of AT-EVs.

Based on the results of *in vitro* experiments, we used a diabetic mouse wound model to explore the effects of AT-EVs *in vivo*. As previously described (Martínez et al., 2019; Wang et al., 2019), we removed the panniculus carnosus to effectively prevent wound contraction without using silicone splints to fix the wound area. To create a moist wound healing environment and maintain AT-EVs *in situ*, they were delivered *via* application of a commercial, cross-linked HA dressing, as described in our previous publication (Liu et al., 2019). *In vivo* experiments demonstrated that the HA + mAT-EVs and HA + cAT-EVs groups had promoted wound healing the most after 14 days without significant difference between the two groups. In contrast, no difference was observed in the wound area between the HA + dAT-EVs and HA + PBS groups, after the same period. The inability of HA + dAT-EVs to promote wound healing, similar to that of the HA + PBS group, may be attributed to contamination of the dAT-EVs with collagenase, during the process of dAT-EV concentration. The collagenase used in this study was a composite of various types of collagenases, which may have contained ingredients unfavorable to wound healing, such as MMP9 (collagenase IV), which has been confirmed as harmful to diabetic wound healing (Muller et al., 2008; Lan et al., 2021). In addition, although our study confirmed the role of AT-EVs in promoting chronic wound healing *in vivo*, the selected animal model was limited to chronic wounds caused by diabetes. Existing studies have shown that EVs could also promote healing of chronic wounds caused by other diseases, such as pressure ulcers, burns, and frostbite injuries (Chen et al., 2019; Willis et al., 2022; Zhang et al., 2022). Further investigation of the therapeutic effect of AT-EVs is necessary to validate its effect.

To realize the effective components of the three types of AT-EVs and reveal the reasons for differences in their effects, miRNA sequencing and bioinformatics analyses were performed, considering that miRNA is one of the most important components of EVs, which plays an important role in regulating cell growth and metabolism (Hill et al., 2013; Abels and Breakefield, 2016; Yu et al., 2016). By comparing the expression profiles of miRNAs contained in human cell-derived EVs in the EVmiRNA database, the overlap rate between AT-EVs and the database was more than 90%, further confirming their cellular origin. However, the database only contained the data of EVs derived from human endothelial cells, fibroblasts, and mesenchymal stem cells. Non-overlapping miRNA expression may be attributed to the fact that AT-EVs were derived from a mixture of other cell types. Sequencing results demonstrated that the three AT-EV types contained certain common miRNAs along with their unique miRNAs. This phenomenon may be attributed to the different outcomes resulting from the different preparation methods. More overlapping miRNAs were identified for dAT-EVs vs mAT-EVs than dAT-EVs vs cAT-EVs and mAT-EVs vs cAT-EVs, indicating that dAT-EVs and mAT-EVs shared more similarities, whereas cAT-EVs differed more from dAT-EVs and mAT-EVs. Considering that adipose tissue was immersed in α-MEM for 36 h and cAT-EVs were prepared from the leach liquor, cAT-EVs theoretically contained more components originating from adipose cells. Contrastingly, dAT-EVs and mAT-EVs may have contained more components originating from non-adipose cells, such as ADSCs and endothelial cells. The exact components and cell origins of the three types of AT-EVs remain unclear, which represents another limitation of this study. In addition, the exact miRNAs, which played a key role in promoting skin wound healing, remain to be further investigated in future.

Summarily, the *in vitro* and *in vivo* results of this study highlighted the obvious advantages of mAT-EVs, derived using the mechanical method. These could be prepared the most swiftly and conveniently in our operation and exhibited the most superior biological effects for diabetic skin wound healing, among the three AT-EVs. The innovation points of this study include our mechanical method, which is presumably the first such method to swiftly and directly prepare EVs with distinguished biological effects for diabetic wound healing, from adipose tissue. Application of this method could rapidly realize the clinical transformation of AT-EVs. Moreover, comparative analysis of the three AT-EVs imparted insight into the characteristics of AT-EVs prepared using different methods, providing objective information for clinical application.

In conclusion, AT-EVs were successfully prepared from adipose tissue by different methods. The derived AT-EVs promoted angiogenesis of HUVECs, as well as cell proliferation and migration of HDFs and HaCaTs, while attenuating the inflammatory response of macrophages *in vitro* and *in vivo*. Comparative analysis results showed that mAT-EVs, derived using the mechanical method, showed the most promise for immediate clinical application and with superior biological effects on delayed diabetic wound healing model.

## Data Availability

The original contributions presented in the study are included in the article/Supplementary Material; further inquiries can be directed to the corresponding authors.

## Abbreviations

MSCs, mesenchymal stem cells; AT-EVs, adipose tissue-derived extracellular vesicles; DMEM, Dulbecco’s modified Eagle’s medium; α-MEM, Eagle’s minimum essential medium alpha-modified; PBS, phosphate buffered saline; FBS, fetal bovine serum; EVs, extracellular vesicles; HA, hyaluronic acid; NTA, nanoparticle tracking analysis; TEM, transmission electron microscopy; HDFs, human dermal fibroblasts; HaCaTs, human immortal keratinocyte cells; HUVECs, human umbilical vein endothelial cells; GO: gene ontology; KEGG, Kyoto encyclopedia of genes and genomes; DAPI, 4′,6-diamidino-2-phenylindole; GAPDH, glyceraldehyde 3-phosphate dehydrogenase.
