# Supplementary material for: Preparation of therapy-grade extracellular vesicles from adipose tissue to promote diabetic wound healing
Source: Front Bioeng Biotechnol. 2023 Mar 23;11:1129187. doi: 10.3389/fbioe.2023.1129187 (PMC10076785; doi:10.3389/fbioe.2023.1129187)
Supplement: Supplementary file 1 [file DataSheet1.docx]

Supplementary Material

Preparation of Therapy-Grade Extracellular Vesicles from Adipose Tissue to Promote Diabetic Wound Healing

Chuqiao Pan^1^†, M.D., Peng Xu^1^†, M.D., Ph.D., Yi Zheng^1^, M.D., Yikai Wang^1^, M.D., Chuhsin Chen^1^, M.D., Shibo Fu^1^, M.D., Ph.D., Zibo Liu^1^, Ph.D., Yahong Chen^1^, M.D., Ph.D., Ke Xue^1^, M.D., Ph.D.,Qimin Zhou^1^*, M.D., Ph.D. and Kai Liu^1^*, M.D., Ph.D.

*** Correspondence:**

* Qimin Zhou

E-mail: qimin.zhou@outlook.com

Tel.: +86-21-2327-1699

Fax: +86-21-5307-8128

* Kai Liu

E-mail: prskailiu@126.com

Tel.: +86-21-2327-1699

Fax: +86-21-5307-8128


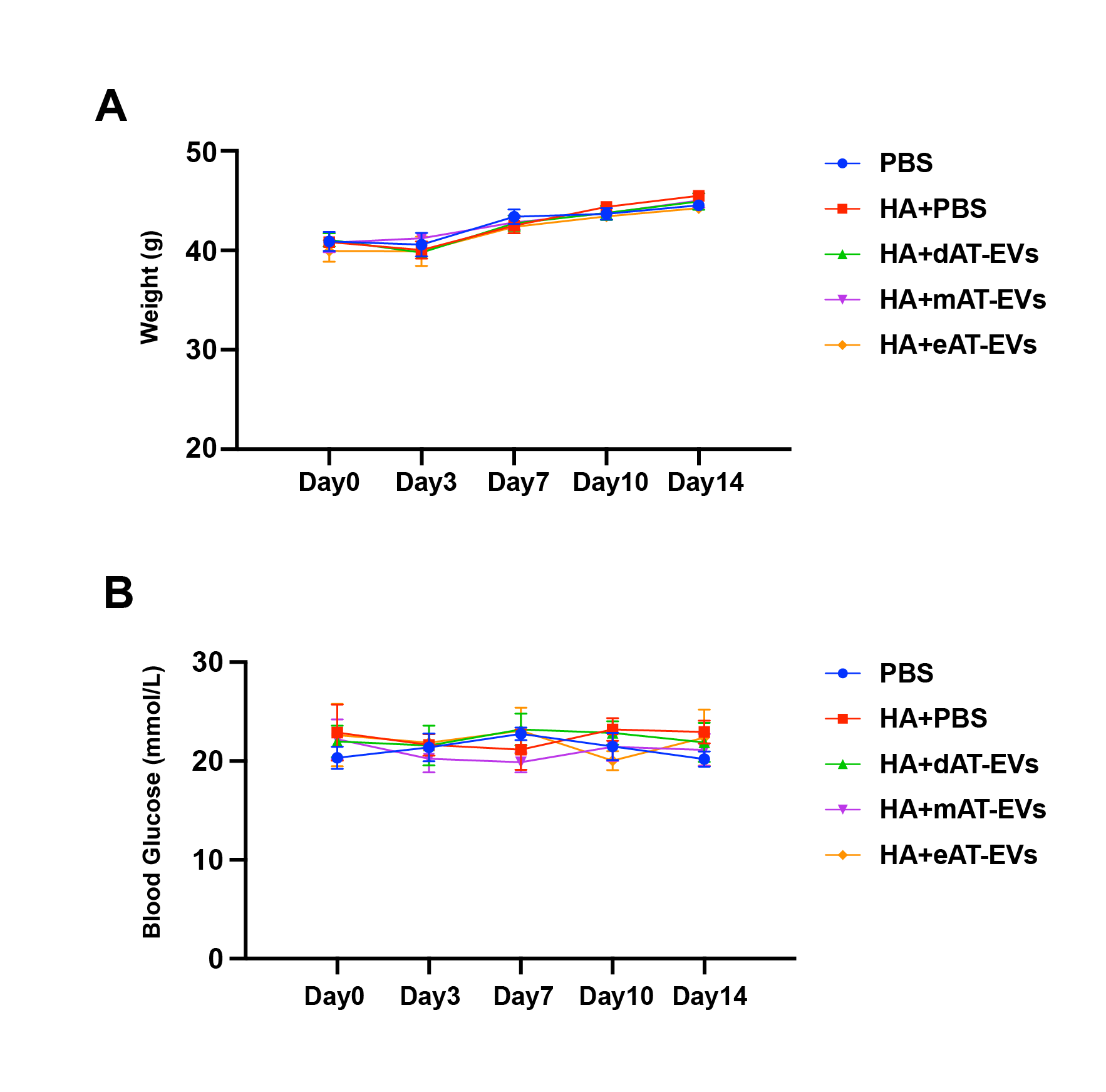


**Supplementary Figure 1. Body weight and blood glucose levels of db/db mice**

1. Body weight of db/db mice on days 0, 3, 7, 10, and 14 (n = 6).
2. Blood glucose levels of db/db mice on days 0, 3, 7, 10, and 14 (n = 6). n.s.: no significant difference observed between groups.

**Table 1. Primers used for qRT-PCR.**

| Genes | Sequences (5’ to 3’) |
| --- | --- |
| Mouse GAPDH | F CCC GTA GAC AAA ATG GTG AA  R TGC CGT GAG TGG AGT CAT AC |
| Mouse IL-6 | F CGG AGA GGA GAC TTC ACA GAG  R ATT TCC ACG ATT TCC CAG AG |
| Mouse TNF-α | F CCA CTC TGA CCC CTT TAC TC  R GCC ATA ATC CCC TTT CTA AGT |
